# Supplementary material for: CIB2, defective in isolated deafness, is key for auditory hair cell mechanotransduction and survival
Source: EMBO Mol Med. 2017 Dec 1;9(12):1711–31. doi: 10.15252/emmm.201708087 (PMC5709726; doi:10.15252/emmm.201708087)
Supplement: Supplementary file 1 — Appendix [file EMMM-9-1711-s001.pdf]

CIB2, defective in isolated deafness, is key for auditory hair cell mechanotransduction and survival

Vincent Michel[1,2,3,*], Kevin T Booth[4,5,*], Pranav Patni[1,2,3,*], Matteo Cortese[1,2,3], Hela Azaiez[4], Amel Bahloul[1,2,3], Kimia Kahrizi[6], Ménélik Labbé[1,2,3], Alice Emptoz[1,2,3], Andrea Lelli[1,2,3], Julie Dégardin[3,7], Typhaine Dupont[1,2,3], Asadollah Aghaie[1,2,3,8], Danuta Oficjalska-Pham[1,2,3], Serge Picaud[3,7], Hossein Najmabadi[6], Richard J Smith[4], Michael R. Bowl[9], Steven D. M. Brown[9], Paul Avan[10], Christine Petit[1,2,3,11], and Aziz El-Amraoui[1,2,3,#]

Table of contents:

Appendix Table S1. Clinical Summary of Patients ………………………………………...………….. 2

Appendix Table S2. CIB2 Mutations and Phenotype Correlation……………………..……………….. 2

Appendix Figure S1: CIB2 immunostaining in wild-type mice, *CIB2*$^{-/-}$ mice, and macaque retinas ………………………………..………………………….…….. 3

Appendix Table S1. Clinical Summary of Patients

Family ID	L-700		Trio-A
**Ethnicity**	Iranian		Palestinian Arab
**Patient ID**	*II.1*	*II.2*	II.1
**Age at Examination**	26	22	12
**Ophthalmologic examination**	NL		NL
**Motor milestone**	NL		NL
**Severity of HL**	Profound		Severe-profound
**Onset of HL**	Prelingual		Prelingual

Italics indicate individuals with fundoscopy images. Age is given in years.

Appendix Table S2. CIB2 Mutations and Phenotype Correlation

Family	Origin	g.DNA	cDNA	Protein	Type	Domain	gnomAD (%)	Conservation		Deleterious				
								GERP	PhyloP	PP2	SIFT	MT	LRT	CADD
**L-700**	Iranian	8423524 G>A	c.34C >T	**p.Gln12***	nonsense		0	C	C	-	-	-	D	38
**Trio-A**	Palestiniar Arab	7840159 3A>T	c.330T >A	**p.Tyr110***	nonsense	EF-2	0	C	C	-	-	-	-	36

Nucleotide numbering: the A of the ATG translation initiation site is noted as +1 using isoform NM_006383 of *CIB2*. NSHL, nonsyndromic hearing loss; C, predicted Conserved; D, predicted Damaging or Deleterious; -, data not available; EF, EF-hand; PP2, PolyPhen-2; MT, MutationTaster.

Appendix Figure S1:

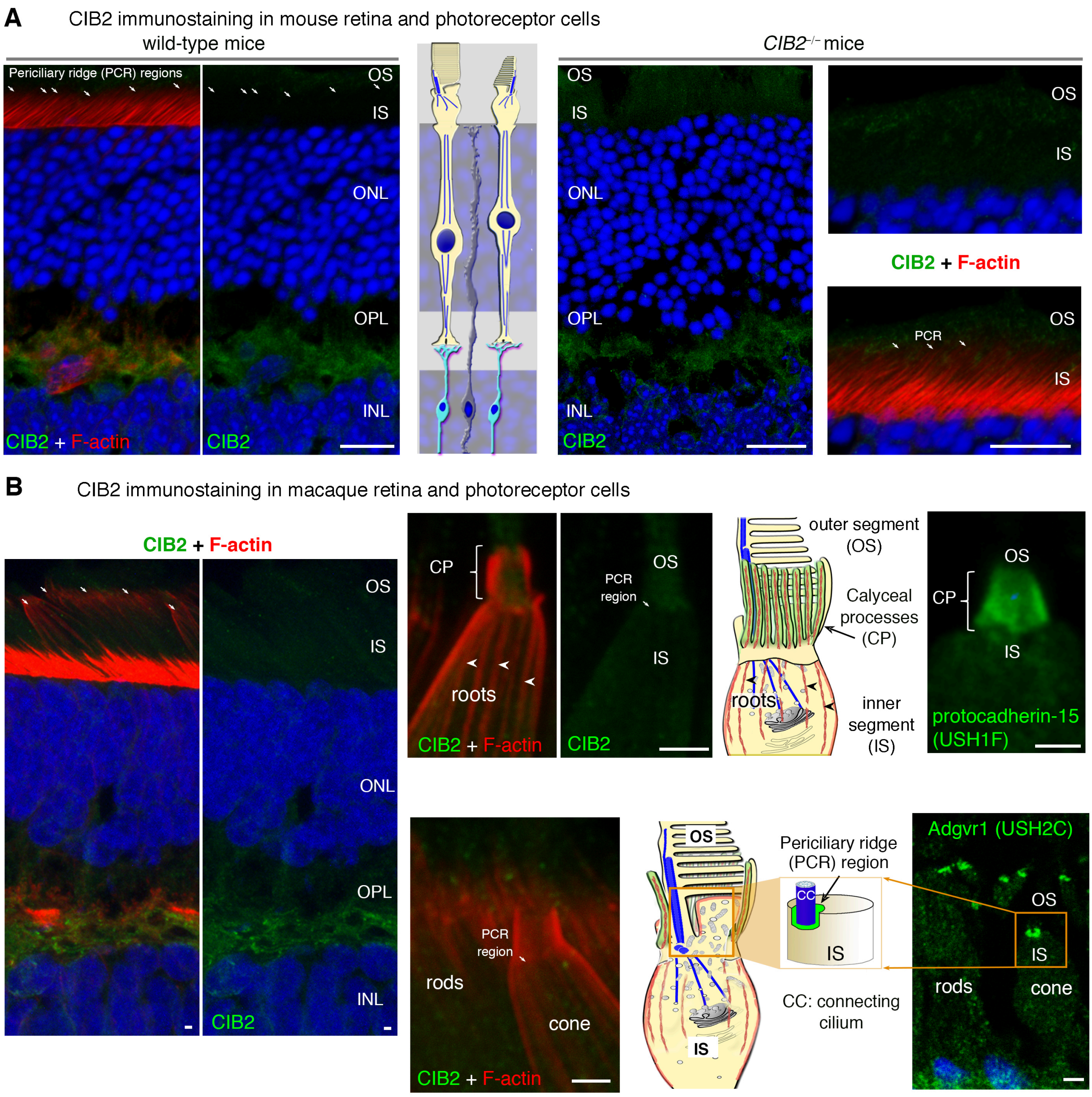

Figure S1: CIB2 immunostaining in wild-type mice, *CIB2*$^{-/-}$ mice, and macaque retinas

(A) Mouse retinas from *CIB2*$^{+/-}$ and *CIB2*$^{-/-}$ mice at 6 months immunostained for CIB2 (green) and F-actin (red) illustrating the absence of specific immunostaining in the photoreceptor cells.

A specific CIB2 immunostaining could not be detected in the inner (IS) and/or outer (OS) segments of rod or cone photoreceptor cells.

(B) Macaque retinas immunostained for CIB2 (green) and F-actin (red). No CIB2 immunostaining was observed in the photoreceptor cells. In the right panels, the immunostaining for protocaherin-15 (USH1F) and Adgvr1 (USH2C) were used to label the calyceal processes, and the periciliary ridge region, respectively. The F-actin staining (red) was used to label the microvillar calyceal processes (CP) and their roots extending downwards in the inner segment.

Scale bars: 10 µm (A) and 2 µm (B). ONL: outer nuclear layer, OPL, outer plexiform layer, INL: inner nuclear layer.
