# Supplementary material for: CIB2, defective in isolated deafness, is key for auditory hair cell mechanotransduction and survival
Source: EMBO Mol Med. 2017 Dec 1;9(12):1711–31. doi: 10.15252/emmm.201708087 (PMC5709726; doi:10.15252/emmm.201708087)
Supplement: Supplementary file 2 — Expanded View Figures PDF [file EMMM-9-1711-s002.pdf]

Expanded View Figures

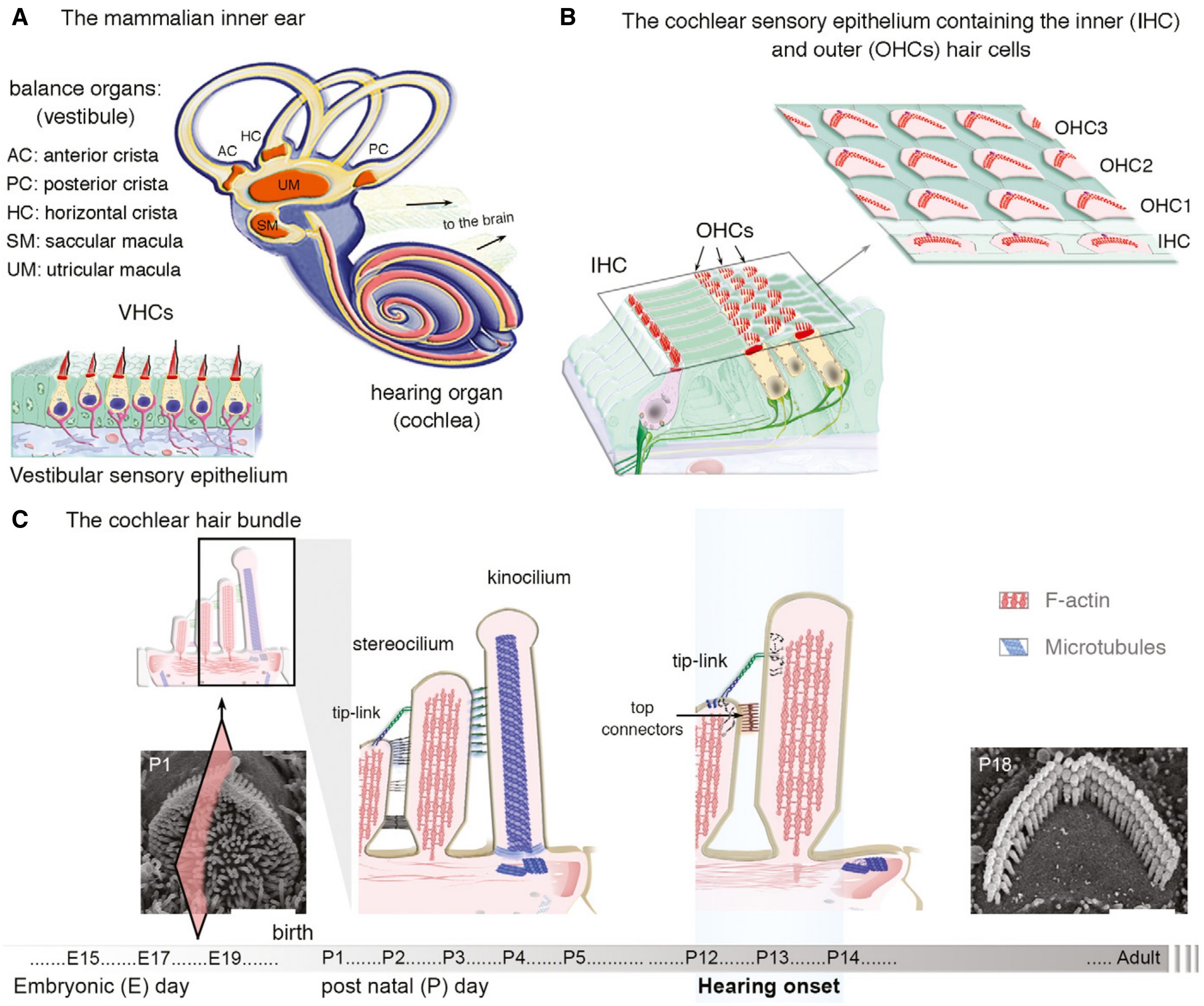

Figure EV1. The hearing and balance inner ear organs, and the morphogenesis of the cochlear hair bundle.

A, B The mammalian inner ear contains five sensory organs of the vestibular apparatus, and a coiled auditory organ called the cochlea (A). There are five vestibular sensory epithelia. The three cristae ampullaris, located in the anterior (AC), horizontal (HC) and posterior (PC) semicircular canals, detect angular acceleration of the head. The two maculae located in the utricle (UM) and saccule (SM) detect horizontal and vertical linear acceleration, respectively. (B) In the auditory sensory epithelium, sensory inner (IHCs) and outer (OHCs) hair cells are organized in a single row and three lateral-side rows, respectively.

C Developing and mature cochlear hair bundles. At the apical surface of hair cells, the hair bundle is made up of an array of actin-filled microvilli, the stereocilia, organized into rows of graded heights that develop under the morphogenetic control of a primary cilium, the kinocilium. During development, a single transient cilium, the kinocilium is located towards the periphery of the hair bundle, attached to adjacent stereocilia in the tallest row. In addition to the tip-link, a fibrous link that gate the MET channels, several types of additional fibrous links hold the stereocilia together and to the kinocilium. In the mature hair bundle, the kinocilium is lost from P10 onwards, and the top connectors in the outer hair cells replace the transient lateral links (Goodyear & Richardson, 1999; Goodyear *et al*, 2005). Scale bars: 2 μm.

 © 2017 Institut Pasteur

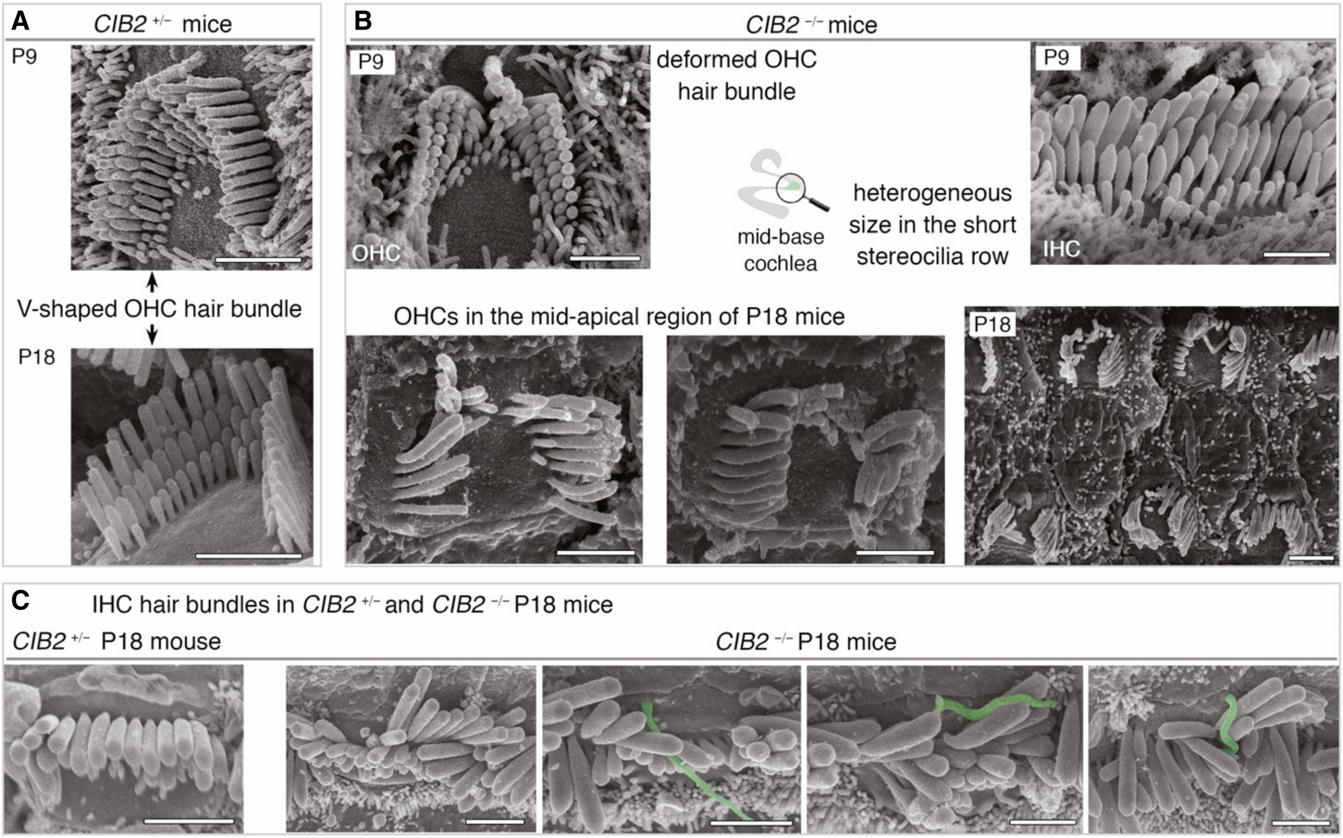

Figure EV2. Abnormal architecture of cochlear hair bundles in *CIB2*$^{-/-}$ mice.

Representative scanning electron microscopy micrographs of the hair bundles in the hair cells of *CIB2*$^{+/-}$ and *CIB2*$^{-/-}$ P9 and P18 mice.

A, B Unlike the cohesive and highly organized V-shaped hair bundle in *CIB2*$^{+/-}$ mice (A), various structural abnormalities are observed in the absence of CIB2, for example horseshoe-like shape, stereociliary shortening, total loss of short row of stereocilia or split bundles (B).

C Several examples of IHC hair bundles in *CIB2*$^{-/-}$ P18 mice illustrating the heterogeneous lengths within IHC stereocilia rows and the persistence in some IHC bundles of the kinocilium (artificially coloured in green).

Data information: Scale bars: 2 μm.

© 2017 Institut Pasteur

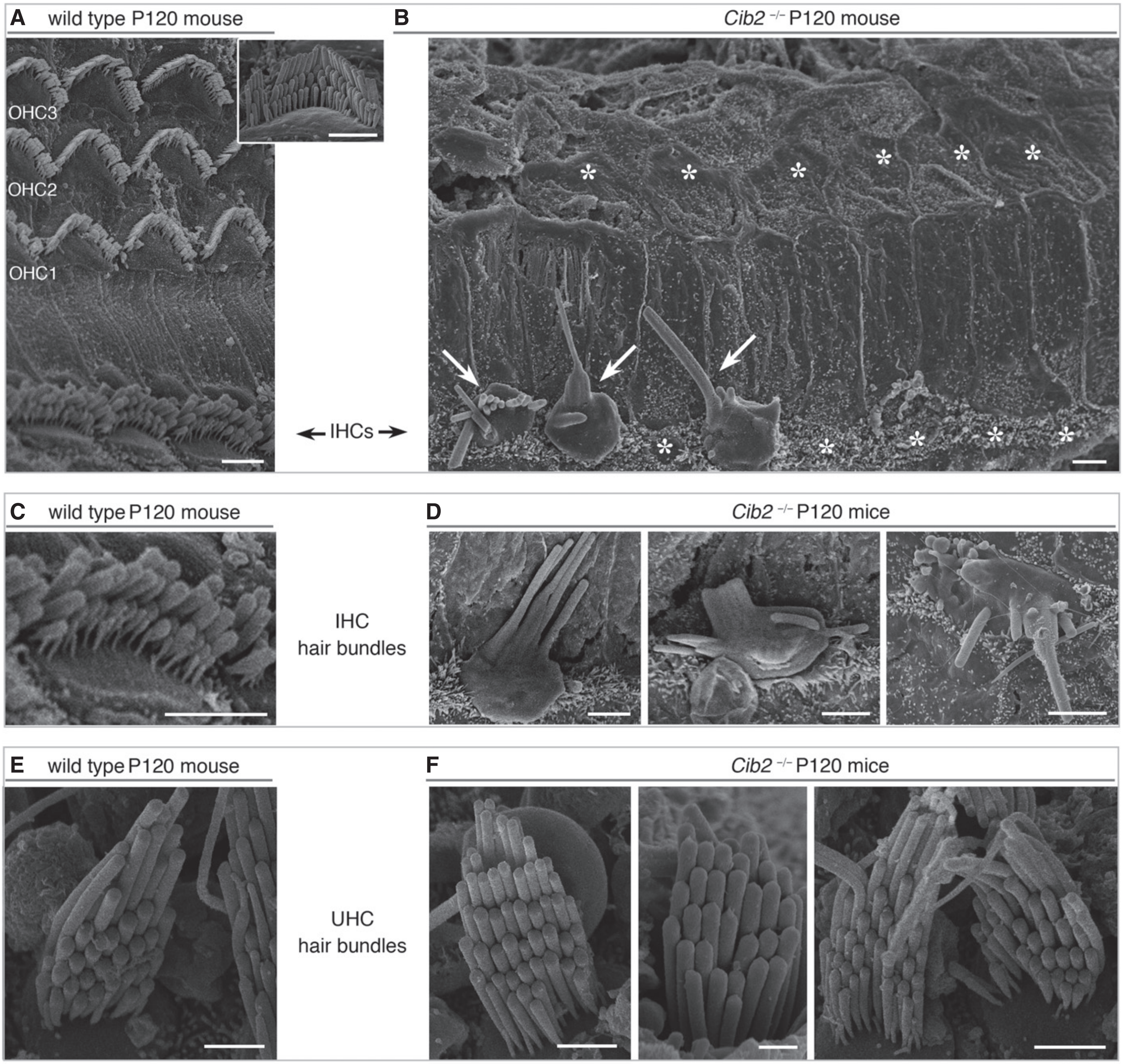

Figure EV3. Loss of the stereocilia and hair bundles in the cochlea of *CIB2*$^{-/-}$ P120 mice.

A–D Top views of cochlear hair cells from *CIB2*$^{+/-}$ and *CIB2*$^{-/-}$ P120 mice. (A, C) The normal architecture of the sensory epithelium and hair bundles from *CIB2*$^{+/-}$ P120 mice is shown for comparison. (B, D) At this stage, most of the IHC and OHC hair bundles, and the apical surface of hair cells, have entirely disappeared (asterisks). The scarce persisting IHC hair bundles (arrows in B) are composed of few stereocilia, often fused or forming bleb-like structures, as shown in the mid-apical region of the cochlea in *CIB2*$^{-/-}$ P120 mice.

E, F Top views of the vestibular utricular hair cells from *CIB2*$^{+/-}$ (E) and *CIB2*$^{-/-}$ (F) P120 mice, showing preserved and normally shaped hair bundles even in the absence of CIB2 (F), contrasting with the severe loss at this stage of the hair bundles in the cochlea (B).

Data information: Scale bars: 2 μm.

 © 2017 Institut Pasteur

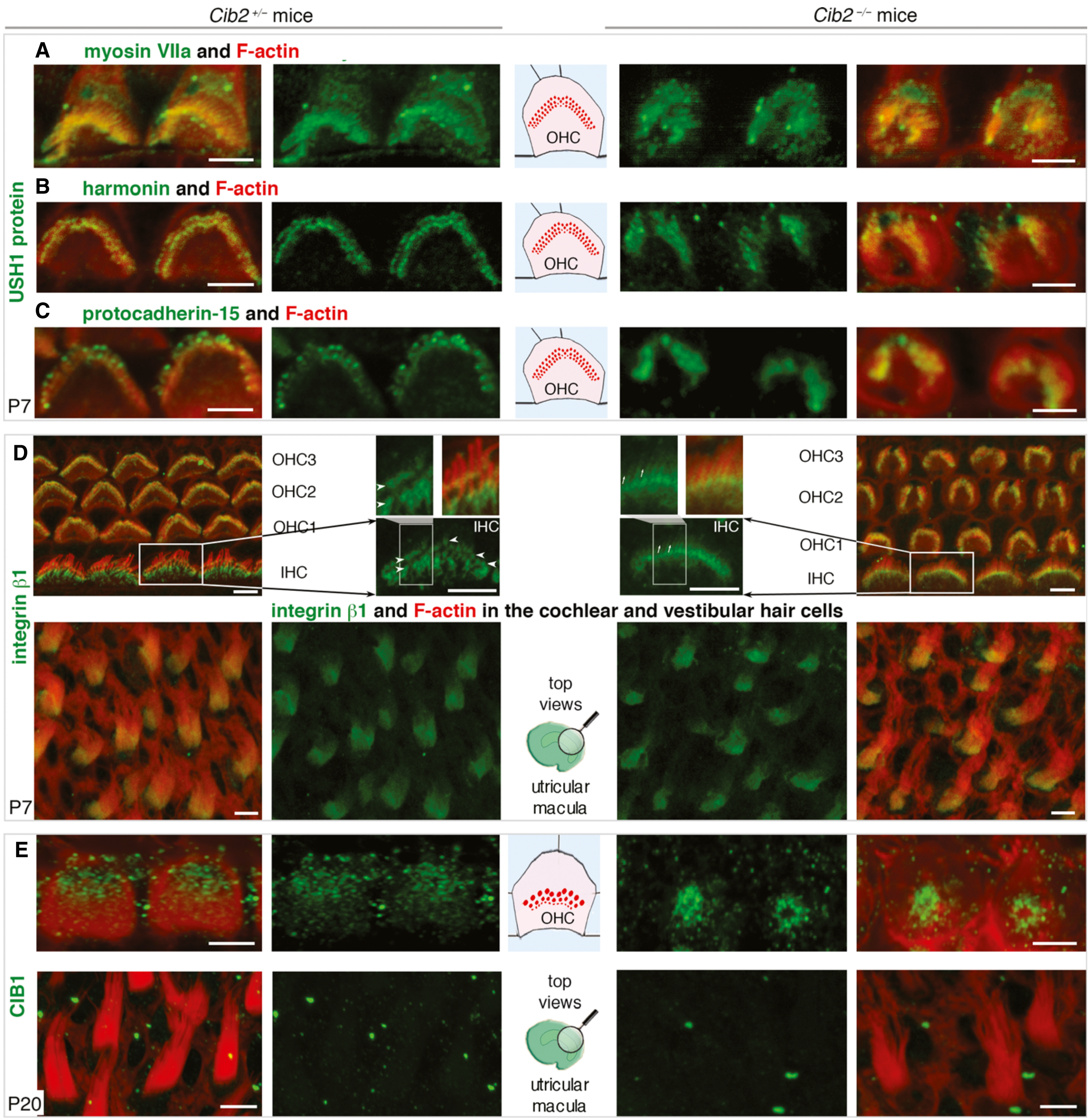

Figure EV4. The distribution of myosin VIIa, harmonin, protocadherin-15, integrins β1 and CIB1 in *CIB2*$^{-/-}$ mice.

A–C Examples of outer hair cells (OHCs) from *CIB2*$^{-/-}$ and *CIB2*$^{+/-}$ P7 mice. There is no evidence for a change in the stereocilia-immunostaining of myosin VIIa (A) harmonin (B) and protocadherin-15 (C) immunostaining (green) in the absence of CIB2.

D For integrin β1, a very minor change could be observed, visible mainly in IHCs (see close-up view). Rather than a restricted staining at the base (arrowheads on the IHC bundle) of the stereocilia, the immunostaining is slightly diffuse, extending along the stereocilia in the absence of CIB2 (arrows). No such change is observed in the hair bundles of vestibular hair cells.

E In *CIB2*$^{+/-}$ P20 mice, CIB1 is located at the apical surface of the hair cells, concentrated at the cell periphery nearby the basal body of the kinocilium. In *CIB2*$^{-/-}$ P20 mice, the CIB1 immunostaining is, instead, found at the apical cell centre of the hair cell.

Data information: Scale bars: 2 µm.

© 2017 Institut Pasteur

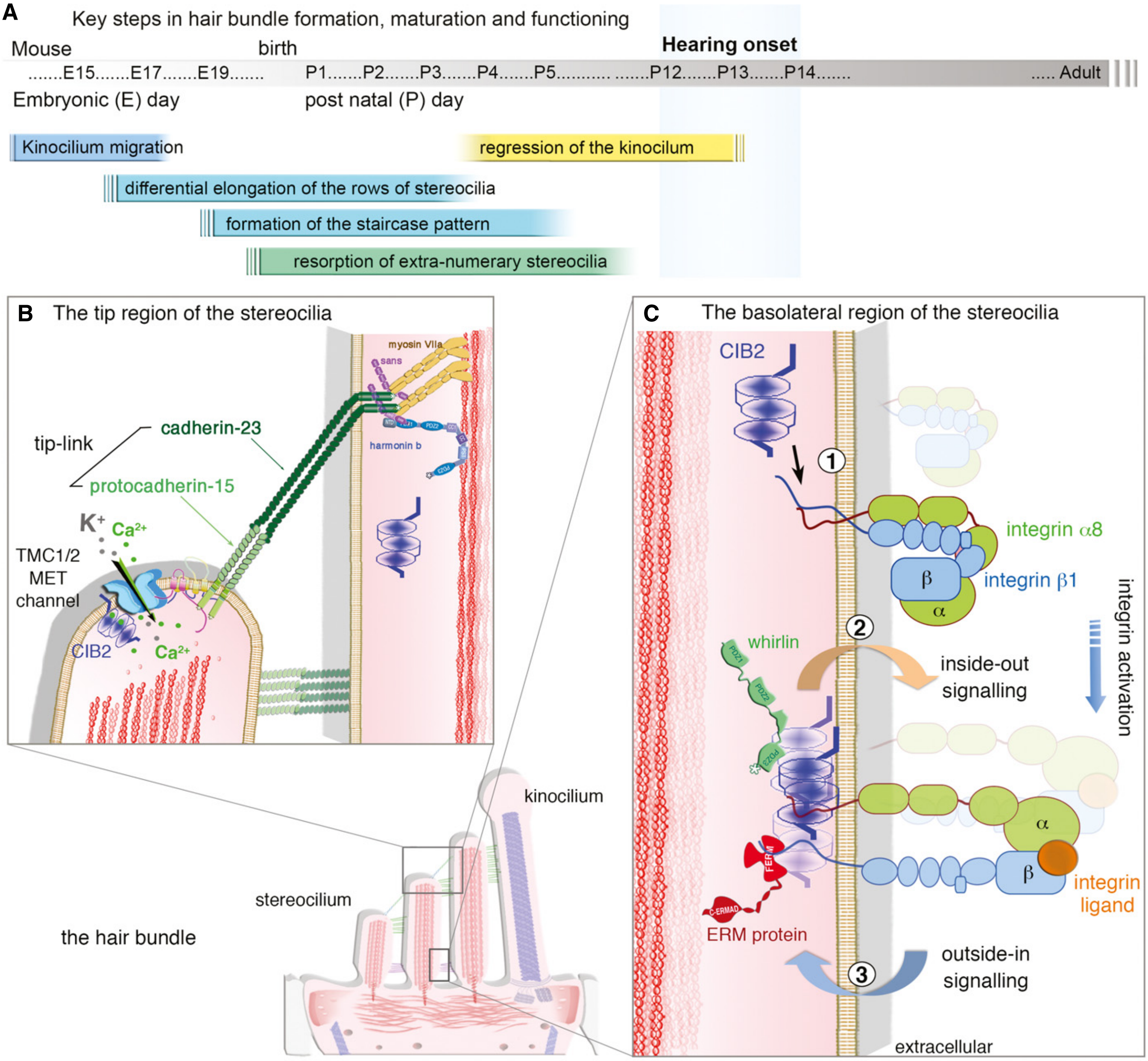

Figure EV5. Schematic representation illustrating the key steps during the morphogenesis of the hair bundle and the role of CIB2 in the apical region of the cochlear hair cells.

A In the mouse differentiating hair cells, the migration of the kinocilium from the cell centre to the cell periphery between E14.5 and E17.5 initiates a differential growth of the stereocilia; those closest to the kinocilium grow faster and longer forming the tallest stereocilia row. The organization into a highly arranged staircase pattern then takes place and is stabilized during the first postnatal week, when the kinocilium and extra-numerary stereocilia disappear and the proper shape of the hair bundle is refined. The molecular and structural organization is set by P12–P14 at hearing onset and is maintained over time to ensure proper transduction of sound.

B, C CIB proteins have been proposed to function as broad regulators of integrin function. (B) At the stereocilia tips, it is yet unknown how lack of CIB2 results in total loss of MET current responses. Possible direct link between CIB2 and the TMC1/2 MET channel complex has been proposed (Giese *et al*, 2017). (C) Because lack of CIB2 has been shown to alter whirlin and integrin α8 staining at the basolateral regions of the stereocilia, it is likely that this protein is part of a signalling platform, probably through multimers of CIB2, influencing the integrin-mediated essential link between the extracellular matrix and the intracellular cytoskeleton of the hair bundle. It has been suggested that CIB proteins activate integrin (1) (Hynes, 2002; Hager *et al*, 2008), in a CIB2-mediated “inside-out” signalling (2) process promoting integrin ligand binding, which in turn would affect potential “outside-in” signalling (3) coupling the extracellular matrix (e.g. the α8 subunit) and intracellular responses in the hair bundle (e.g. whirlin and/or FERM proteins). Such a crosstalk might be essential to maintain the shape and stereocilia integrity of the mature functioning hair bundles.

 © 2017 Institut Pasteur
